# ECHO project in atopic dermatitis in Argentina: An innovative strategy to reach underserved areas with up to date knowledge, first year of experience^⋆^

**DOI:** 10.1016/j.abd.2021.09.006

**Published:** 2022-05-25

**Authors:** Paula C. Luna, María Emilia Debernardi, Cristina Mariela Echeverría, María Valeria Angles, Luis D. Mazzuoccolo

**Affiliations:** a Dermatology Department, Hospital Alemán, Ciudad Autónoma de Buenos Aires, Argentina; b Instituto de Rehabilitación Psicofísica, Ciudad Autónoma de Buenos Aires, Argentina; c Dermatology Department, Hospital Italiano de Buenos Aires, Ciudad Autónoma de Buenos Aires, Argentina

**Keywords:** Atopic dermatitis, Medical education, Mentoring, Information technology

## Abstract

**Background:**

The ECHO® (Extension for Community Healthcare Outcomes) project is a model of distance medical education developed in the United States to support health professionals in the management of patients with complex diseases. Since 2019, it has been implemented in atopic dermatitis (AD) in Argentina. The program consists of the periodic presentation of clinical cases by videoconference, virtual classes, and a permanently available open chat between professionals in charge of patients with AD and a group of experts.

**Objective:**

The objective of this study was to analyze the impact of the ECHO Project AD on the medical knowledge and medical skills of Argentinian health professionals when treating patients with AD.

**Methods:**

A survey was carried out among the participants in order to evaluate the impact of the program on the care of patients with AD.

**Results:**

ECHO Project AD revealed a significant improvement in the management of patients with AD. The program contributed to the interpretation and use of severity scores, use of phototherapy, and management and prescription of both classic and innovative topical and systemic treatments.

**Study limitations:**

The reduced number of participants and the short period of time. The answers of the survey may be biased by the enthusiasm of the participants.

**Conclusions:**

The ECHO project is an educational tool that enhances the medical skills of doctors and institutions, in which a climate of a partnership comes first and the participants look forward to learning from experiences, successes, and mistakes from one another, producing a scientific hub in constant evolution.

## Introduction

Project ECHO® (Extension for Community Healthcare Outcomes) is an innovative guided-practice model whose main purpose is to support health providers in the management of patients with chronic, prevalent, and complex diseases worldwide, thereby reducing disparities between under-served and remote areas and large urban centers. It takes place through knowledge-sharing teleconferences between physicians and specialist mentors at academic medical centers.^1^, ^2^

The program was developed at the University of New Mexico (UNM) School of Medicine to generate knowledge-sharing hubs between health providers and support professionals in remote areas in the management of patients with hepatitis C infection. Since it proved to generate knowledge networks and improve both quality and access to health providers, it has spread globally and nowadays includes 960 different programs in 45 countries around the world.^3^ Since 2015 the authors have implemented and successfully conducted the Project ECHO Psoriasis in Argentina. It was the first program fully developed for a dermatologic condition.^4^ Four years later, at the beginning of 2019, Project ECHO Hidradenitis was launched, and towards the end of 2019, the authors started Project ECHO Atopic Dermatitis.

Atopic Dermatitis (AD) is a common inflammatory skin disorder characterized by recurrent eczematous lesions and intense itch, which significantly affects the quality of life of patients and their families. AD Is often associated with a familiar or personal history of atopy. The incidence of AD is increasing worldwide. Although in our country the authors do not have official statistics, globally, it affects up to 20% of children and 10% of adults, 10% to 20% of which suffer from a severe disease.^5^, ^6^, ^7^

Although diagnosing AD in pediatric patients usually doesn't present major difficulty, in adults, there might be up to a 10-year diagnosis delay. There is also an important asymmetry regarding diagnosis and treatment between Buenos Aires City and the Greater Buenos Aires area compared to other provinces (Data belonging to AEPSO/ ADAR SURVEY 2020. https://www.aepso.org/da/, not yet published).

On the other hand, the great geographic extension of Argentina and the high concentration of professionals in large urban centers make it difficult for patients to access health specialists. In turn, 40.7% of patients in Argentina are unsatisfied with the treatment they receive (Data belonging to AEPSO/ ADAR SURVEY 2020. https://www.aepso.org/da/, not yet published).

Lastly, both the dynamic knowledge about the pathophysiology of AD and the development of therapeutic molecules make the ECHO project a valuable tool for physicians to receive "on-demand" education and, finally, to acquire good clinical practices and improve the quality of health care. In this article, the authors describe the present study’s results when replicating the ECHO model in AD in Argentina.

## Materials and methods

The authors reproduced the ECHO AD program in Argentina on the basis of the ECHO psoriasis project, for which one of the mentors had received training and signed an agreement between the UNM and its institution for the development of the dermatology program.

Dermatologists, dermatologist in training (residents and fellows), and other related specialists (pediatricians and allergists) from the Society of Pediatric Dermatology for Latin America database and ECHO Psoriasis program participants were invited to participate via WhatsApp® or email.

Information about the project was provided: its objectives and reach, the use of the teleconference platform, and the modality of presentation of clinical cases.

Scheduled Meetings were carried out through 90 minutes duration videoconference (via Zoom® platform) monthly.

Before each meeting, the clinical cases to be presented were scheduled. Experts moderated each session, and different physicians presented the clinical cases in the pre-established format (Power Point®). The discussion was then open to all participants, exchanging their clinical views and finally reaching an agreement about the way to proceed.

Due to treatment failures or adverse effects, participants were able to resubmit the same cases for reevaluation.

On average, there were 2 or 3 cases per meeting.

In addition, during every meeting, there was a scheduled short update lecture regarding topics related to AD. (Pathophysiology, severity scores, emerging treatments, comorbidities, etc.).

A WhatsApp® group was created in order to send queries that required quick resolution and could not wait until the next meeting or to share information related to the pathology on an educational purpose.

After 12 meetings, the professionals answered an anonymous survey in order to evaluate the educational results of the project on their medical skills and the impact of the program on their daily practice (Table 1). Descriptive quantitative statistics were used for its analysis.

**Table 1:** Survey sent to participants at the end of the 12 meetings.

Survey sent to participants at the end of the 12 meetings.
- In which province do you practice?
- Years of practice in the specialty.
- Specialty.
- Do you participate in other ECHO programs?
-Have you participated in ECHO AD Zoom® meetings?
- Are you joining the ECHO AD chat?
- How do you think ECHO AD has impacted on your understanding and general management of AD patients?
(It got much better / It got a little bit better / It stayed the same / It got a little worse / It got worse)
- How do you think ECHO AD has impacted your understanding of the pathophysiology of AD?
(It got much better / It got a little bit better / It stayed the same / It got a little worse / It got worse)
- How do you think ECHO AD has impacted on your ability to diagnose AD?
(It got much better / It got a little bit better / It stayed the same / It got a little worse / It got worse)
- How do you think ECHO AD has impacted your knowledge of the severity scores?
(It got much better / It got a little bit better / It stayed the same / It got a little worse / It got worse)
- Did you use the DA severity scores before ECHO AD?
- How often do you plan to use the AD severity scores in the near future?
- Did you know before the program the role that the use of emollients had in AD?
- Did ECHO AD improve your understanding of the importance of emollients in AD?
- Did ECHO AD improve your knowledge about the different topical drugs and possible application schemes? (Proactive treatment, weekend therapy, etc.).
- Did ECHO AD improve your knowledge about the use of phototherapy in AD?
- Did ECHO AD improve your knowledge about the use of systemic treatments in AD?
- How likely are you to use classical systemic treatments in the short term?
- Did ECHO AD improve its knowledge about the use of Dupilumab treatment in AD?
- How likely are you to use Dupilumab in the short term?
- How much would you recommend ECHO AD to a colleague?
1 (No way) ‒ 5 (sure).

## Results

A total of 12 meetings were held between November 2019 and November 2020. A total of 28 patients were presented for discussion in these sessions. In addition, there were 35 cases of patients who required urgent discussion in the WhatsApp® group, which ran in parallel and permanently.

**Figure 1:**
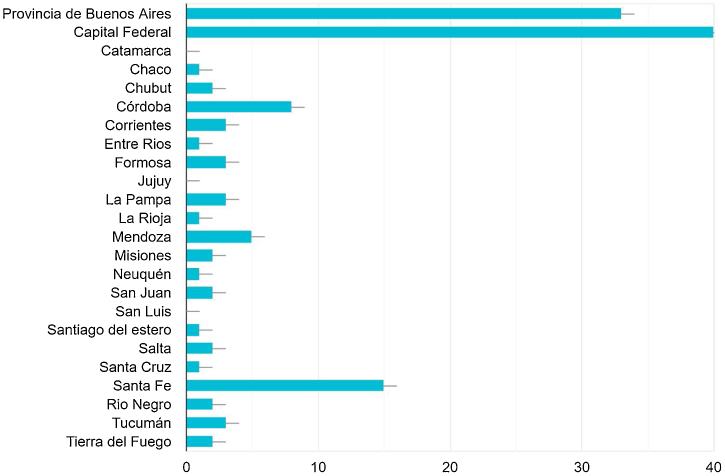
Distribution of participants according to the different provinces and Buenos Aires city.

A total of 217 physicians (dermatologists, allergists, and residents of these specialties) from 20 provinces (all except San Luis, Jujuy, and Catamarca) and Buenos Aires city conform the ECHO DA hub (Fig. 1).

**Figure 2:**
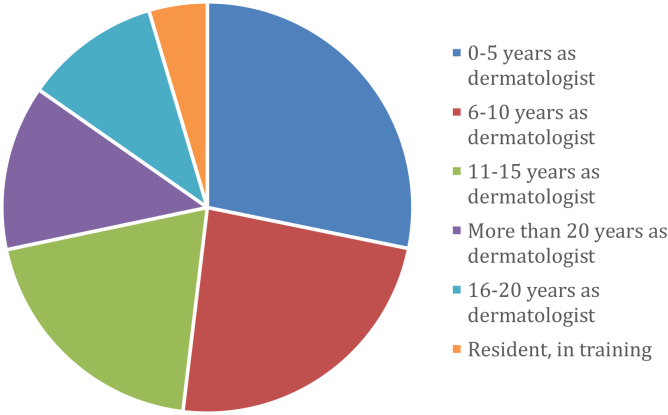
Distribution of participants according to years of experience in the practice of the specialty.

**Figure 3:**
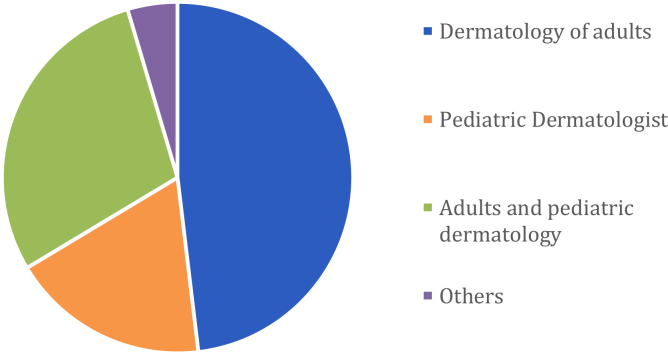
Distribution of particip1ants according to specialty.

More than a half of the participants had less than 10-years as a dermatologist (28.2% with less than 5-years and 23.7% between 6 and 10 years), 19.8% between 11 and 15 years, 10.7% between 16 and 20, 13% more than 20 years and a small percent were residents 4.6% (Fig. 2). The vast majority were adults dermatologists, followed by adult and pediatric' dermatologists and pediatric' dermatologists (Fig. 3). 74.2% of the professionals participated in other ECHO project (71.7% ECHO Psoriasis and 35.9% ECHO Hidradenitis Suppurativa). 90% of the participants were able to attend at least one virtual meeting, and 95.4% participated in the chat (of which the majority read but did not write).

Regarding the impact of ECHO DA on the medical skills of the participants, 72% believed that it greatly improved the understanding and general management of patients with AD, 93% said that it had a positive impact on the understanding of pathophysiology, 78.6% improved their diagnostic capacity and 88.5% their knowledge of clinical severity scores. On the other hand, more than 85% of the professionals believed that ECHO DA contributed to improving their knowledge about the different topical drugs and possible application schemes, and 71.5% of their knowledge about phototherapy. Regarding the use of systemic treatments, 62.5% thought that it was a great improvement and 23.4% moderate improvement, but in reference to the use of dupilumab, 69.2% thought that the program contributed greatly to the management of this medication. Lastly, more than 98% would recommend ECHO DA to their colleagues.

## Discussion

Through continuous medical training and the support of doctors who are in remote places with less access to it, the ECHO project allows patients with complex diseases to receive quality care in their places of origin, avoiding the need to travel to large urban centers.

This innovative telementoring tool has spread throughout the world, and today there are 960 ECHO programs in 45 countries.^3^

By sharing clinical cases with other colleagues and experts, the project contributes to improving the medical care of a particular patient, but also to conform to a scientific knowledge hub that might be considered an educational strategy framed within ICT (Technology of the Information and Communication).

In this study, the authors describe our initial experience in replicating the ECHO project in AD.

Due to the great geographic extension of the authors’ country and the asymmetry in the distribution of specialists in the different provinces, the project takes an important place in Argentina, which is reflected in the participation of doctors from 20 provinces and Buenos Aires city.

By means of a survey, the authors could measure the impact of the project on the medical skills of the participants. The authors conclude it was very positive, achieving in most cases the expansion of knowledge of therapeutic tools for patients with AD.

After the course of a year, the authors have managed to meet the initial objective of the program: to reduce possible asymmetries in the training of physicians through medical education and remote-assisted clinical practice.

In a second instance, the authors will assess the impact of the program on the exercise of good medical practices and the impact on the quality of patient care, since, although it is inferred that more education and better tools will result in better care, that aspect was not objectively measured in this first study.

Taking into account that a great part of the first year of the program took place during the COVID-19 pandemic, in the authors’ opinion, the ECHO project was particularly important to reduce medical isolation and guarantee good quality medical care to our patients with AD.

## Conclusion

The ECHO project is an educational tool that enhances the medical skills of doctors and institutions, in which a climate of a partnership comes first, and the authors look forward to learning from experiences, successes, and mistakes of our own and other colleagues, conforming to a scientific hub in constant movement.

## Financial support

None declared.

## Authors' contributions

Paula C. Luna: Approval of the final version of the manuscript; critical literature review; data collection, analysis, and interpretation; effective participation in research orientation; intellectual participation in propaedeutic and/or therapeutic management of studied cases; critical manuscript review; preparation and writing of the manuscript; statistical analysis; study conception and planning.

María Emilia Debernardi: Approval of the final version of the manuscript; critical literature review; data collection, analysis, and interpretation; effective participation in research orientation; intellectual participation in propaedeutic and/or therapeutic management of studied cases; critical manuscript review; preparation and writing of the manuscript; statistical analysis; study conception and planning.

Cristina Mariela Echeverría: Approval of the final version of the manuscript; critical literature review; data collection, analysis, and interpretation; effective participation in research orientation; intellectual participation in propaedeutic and/or therapeutic management of studied cases; critical manuscript review; preparation and writing of the manuscript; statistical analysis; study conception and planning.

María Valeria Angles: Approval of the final version of the manuscript; critical literature review; data collection, analysis, and interpretation; effective participation in research orientation; intellectual participation in propaedeutic and/or therapeutic management of studied cases; critical manuscript review; preparation and writing of the manuscript; statistical analysis; study conception and planning.

Luis D. Mazzuoccolo: Approval of the final version of the manuscript; critical literature review; data collection, analysis, and interpretation; effective participation in research orientation; intellectual participation in propaedeutic and/or therapeutic management of studied cases; critical manuscript review; preparation and writing of the manuscript; statistical analysis; study conception and planning.

## Conflicts of interest

Luna Paula Carolina has served as scientific adviser/received honoraria from Abbvie, Eli Lilly and company, Janssen, Novartis, Pfizer and Sanofi Genzyme.

Debernardi María Emilia has received an educational grant from Pfzier.

Echeverría Cristina Mariela has served as scientific adviser / received honoraria from Abbvie, Amgen, Eli Lilly and company, Janssen, Novartis, Pfizer and Sanofi Genzyme.

Angles María Valeria has served as scientific adviser / received honoraria from Abbvie and Sanofi Genzyme.

Mazzuoccolo Luis Daniel has served as scientific adviser / received honoraria from Abbvie, Amgen, Eli Lilly and company, Janssen, Novartis and Sanofi Genzyme.
